# Molecular Prognostic Factors for Distant Metastases in Premenopausal Patients with HR+/HER2− Early Breast Cancer

**DOI:** 10.3390/jpm11090835

**Published:** 2021-08-25

**Authors:** Hua Ni, Jörg Kumbrink, Doris Mayr, Alina Seiler, Friederike Hagemann, Tom Degenhardt, Sabine Sagebiel, Rachel Würstlein, Ronald Kates, Nadia Harbeck, Tanja K. Eggersmann

**Affiliations:** 1 Breast Center, Department of Gynecology and Obstetrics and Comprehensive Cancer Center (CCC)-Munich, LMU University Hospital, 80337 Munich, Germany; H.Ni@campus.lmu.de (H.N.); alina.seiler@web.de (A.S.); Friederike.Hagemann@med.uni-muenchen.de (F.H.); Tom.Degenhardt@med.uni-muenchen.de (T.D.); Rachel.Wuerstlein@med.uni-muenchen.de (R.W.); Nadia.Harbeck@med.uni-muenchen.de (N.H.); 2 Faculty of Medicine, Institute of Pathology, Ludwig-Maximilians-University of Munich (LMU), 80337 Munich, Germany; Joerg.Kumbrink@med.uni-muenchen.de (J.K.); Doris.Mayr@med.uni-muenchen.de (D.M.); Sabine.Sagebiel@med.uni-muenchen.de (S.S.); 3 REK Consulting, 83624 Otterfing, Germany; Ronald.Kates@t-online.de; 4 Department of Gynecological Endocrinology and Reproductive Medicine, University Hospital of Schleswig-Holstein, 25538 Luebeck, Germany

**Keywords:** breast cancer, distant metastasis, premenopausal, hormonal receptor positive, HER2 negative, mTORC1, ROR, molecular markers, expression signature tests, prognostic factors

## Abstract

Molecular factors that drive metastasis in premenopausal patients with hormone receptor positive (HR+), human epidermal growth factor receptor 2 negative (HER2−), early breast cancer (EBC) are largely unknown. To identify markers/signatures contributing to metastasis, we analyzed molecular changes in tumors from premenopausal patients who developed metastasis (M1) and who did not (M0). Ninety-seven premenopausal patients with HR+/HER2− EBC were included (M1, *n* = 48, median distant metastasis-free survival (DMFS): 54 (7–184) months; M0, *n* = 49, median follow-up: 149 (121–191) months). Gene expression profiling on tumor RNA (Breast Cancer 360^TM^ panel, Nanostring) was performed, followed by comprehensive bioinformatic and statistical analyses. Significantly enhanced ROR (risk of recurrence) scores and reduced signature scores of PGR (progesterone receptor), claudin-low, and mammary stemness were determined in M1. These differences were significantly associated with shorter DMFS in univariate survival analyses. Gene set enrichment analysis showed an enriched mTORC1 pathway in M1. Moreover, a metastasis signature of 19 differentially expressed genes (DEGs) that were DMFS-related was defined. Multivariate analysis including the four signatures, 19 DEGs, pN, and pT status, identified *LRP2*, *IBSP,* and *SCUBE2* as independent prognostic factors. We identified prognostic gene signatures and single-gene markers for distant metastasis in premenopausal HR+/HER2− EBC potentially applicable in future clinical practice.

## 1. Introduction

Breast cancer has the highest incidence and mortality rate among all cancer types in women. For young women under 50, breast cancer has an incidence rate of 20% and a mortality rate of 4%, ranging first globally [1]. As one of the most promising and encouraging concepts in this century, precision medicine or personalized medicine is crucial for optimizing the clinical management of breast cancer patients and has been a research hot spot for decades [2,3,4].

During the past two decades, rapid development and application of high throughput screening technologies have further revealed the complexity of breast cancer: breast cancer is not a single disease but consists of various subtypes that require specialized management [5,6]. Since the first attempts to subtype breast cancer by gene expression patterns [5], clinical diagnosing and treatment routines have been dramatically influenced and even transformed by molecular subtyping methods and gene-level research [4]. Intrinsic subtypes including luminal A, luminal B, HER2 (human epidermal growth factor receptor 2)-enriched (HER2-E), basal-like, claudin-low, and normal-like are widely referred to while predicting prognosis and selecting treatment [4]. Moreover, molecular risk-stratifying tools such as Oncotype DX^®^, MammaPrint^®^, Prosigna^®^, and EndoPredict^®^ are now essential supplements in the clinical treatment planning procedure [7,8].

Nonetheless, the diversity of breast cancer (BC) has not yet been fully explored and requires more detailed research to prolong the survival of each individual. Guidelines for hormone receptor-positive (HR+), HER2-negative (HER2−) breast cancer, which constitutes the majority of diagnosed early breast cancer (EBC), generally mandate adjuvant treatment after surgery [4]. Typical treatment regimens consist of endocrine therapy with or without radiotherapy and/or chemotherapy [4]. HR+/HER2− EBC is a potentially curable disease, yet nearly 50% of the patients with HR+/HER2− EBC are resistant to endocrine therapy [9], and around 30% of patients develop distant metastasis; metastatic breast cancer is at present an incurable disease associated with severely limited overall survival [10]. Elucidating molecular drivers of distant metastasis is of intense clinical interest and would benefit the individualization of both diagnosis and treatment.

Breast cancer in premenopausal patients generally has a worse prognosis than in post-menopausal patients, especially for hormone receptor-positive tumors [11,12]. As to the cause, research suggests that breast cancer in young patients is more commonly associated with endocrine resistance [13], is often of higher grade [14], has more aggressive molecular patterns [9], and could be of unique biology that requires novel therapeutic methods [15,16,17]. Considering the unique molecular patterns of breast cancer [15,17], specific molecular research on premenopausal patients with HR+/HER2− EBC is necessary.

The present analysis focused on premenopausal patients with HR+/HER2− EBC and aimed to refine the knowledge of molecular characteristics associated with distant metastasis in this group of patients. 

## 2. Materials and Methods

### 2.1. Study Population

In this retrospective study, a total of 278 premenopausal patients with HR+/HER2− invasive EBC who underwent surgery at the LMU (Ludwig-Maximilians-University of Munich, Munich, Germany) breast center between 1998 and 2012 were included in a matched cohort design.

During the screening and enrollment process, 124 patients were lost to follow-ups, and 57 did not have the minimum tumor amount for RNA extraction. We identified 97 premenopausal patients with sufficiently long follow-up, 48 patients of whom with distant metastases (M1) and 49 patients without (M0; shortest follow-up was 10 years).

Clinical information, including age, tumor size, side, grade, histological type, stage, and treatment are presented in Table 1. HR and HER2 scoring were performed during routine diagnostics at the Institute of Pathology of the LMU (Germany). HR+ was defined as ER and/or PR with IHC (immunohistochemistry) scores of at least 10/100 following the ICA (International Council on Archives) standard or 1/12 according to the IRS (immunoreactive score) standard. HER2 was considered negative (HER2−) with IHC scores of 0–1+ or IHC 2+ and negative in FISH (fluorescence in situ hybridization) analysis. Patients received treatment regimens based on guidelines and according to investigators’ choices.

### 2.2. Ethics Approval and Consent

The study received approval from the Institutional Review Board of the Ludwig-Maximilians-University of Munich (LMU) (Germany) (Number: 19–745).

### 2.3. Gene Expression Profiling

Tumor samples obtained during the initial tumor resection were used for gene expression profiling. Histopathological diagnosis and classification were elaborated by two experienced pathologists at the Institute of Pathology of the LMU (Munich, Germany). Sections from formalin-fixed paraffin-embedded (FFPE) tissue specimen were prepared followed by hematoxylin-eosin staining of one slide. Microdissection was performed on areas with a minimum percentage of 50% tumor cells from subsequent unstained sections and used for RNA preparation. Detailed information for every tumor sample can be found in Table S1.

Total RNA was extracted from 4 to 8 sections of FFPE tissue sections using the miRNeasy kit (QIAGEN, Hilden, Germany) according to the manufacturer’s instructions. RNA yield and purity were assessed using the NanoDrop ND-1000 spectrophotometer (NanoDrop Technologies, Wilmington, DE, USA). Samples were analyzed on a nCounter^®^ FLEX system (Nanostring technology, Seattle, WA, USA) with the Breast cancer 360^TM^ panel, which includes 776 genes across 23 key breast cancer pathways and processes. Briefly, 250 ng of total RNA was hybridized for 18 h at 65 °C with the NanoString code set and nCounter Prep Station loading as well as expression quantification with the nCounter Digital Analyzer was performed as recommended by the manufacturer.

### 2.4. Gene and Signature Expression Analysis

Expression data quality control was performed using the default nSolver v4.0 software settings and by analyzing reference genes, positive/negative controls, total counts, and binding densities in each sample. Twelve samples had to be excluded due to insufficient quality; 85 samples were analyzed further. Genes in the TIS (tumor inflammation signature) signature were normalized using the ratio of the expression value to the geometric mean of the housekeeper genes used only for the TIS signature. Genes in the PAM50 signature were normalized using the ratio of the expression value to the geometric mean of the housekeeper genes used only for the PAM50 signature. All other genes were normalized using a ratio of the expression value to the geometric mean of all housekeeping genes on the panel.

The detailed methods for PAM50 subtyping and ROR scoring were published previously [18]: PAM50 subtype calls were the result of a 3-step algorithm to identify luminal A, luminal B, HER2-enriched, and basal-like prototypical breast cancer subtypes. Each patient received a score for each subtype; the highest score decided the subtype call. ROR (risk of recurrence) scores were the result of a multi-step algorithm and scaled to lie between 0 and 100. The scores of 46 BC360 signatures were calculated for each sample based on the gene expression data using the BC360 algorithm by Nanostring.

### 2.5. Differential Expression Analysis

Differential expression was fit on a per-gene or per-signature basis using a linear model for analyses without a blocking factor. The statistical model used the expression value or signature score as the dependent variable and fit a grouping variable as a fixed effect to test for differences in the levels of that grouping variable. *p*-values were adjusted within each analysis (gene or signature) and on the grouping variable level difference *t*-test using the Benjamini and Yekutieli False Discovery Rate (FDR) adjustment to account for correlations amongst the tests. Unadjusted *p*-values were presented where no significant adjusted *p*-value was observed. All models were calculated using the limma package in Reference [19]. Differentially expressed genes (DEGs) were selected based on the following standard: *p* < 0.05 and abs(log2 FC_single gene_) > (mean(abs(log2 FC_all genes_)) + 2 SD(abs(log2 FC_all genes_))). Heatmaps and volcano plots of Signatures/DEGs were created with the pheatmap and “ggplot2” functions in R, respectively. GO (gene ontology) and KEGG (Kyoto Encyclopedia of Genes and Genomes) analyses of DEGs were performed by using the Clusterprofiler package in Ref [20]. STRING (search tool for the retrieval of interacting genes/proteins) analysis was conducted to investigate the functional interactions of the identified DEGs [21].

GSEA (Gene Set Enrichment Analysis) was performed with the GSEA 4.1.0 software utilizing “h.all.v7.4.symbols.gmt” datasets (hallmarks) following the instructions [22]. FDR q-value < 0.25 was considered significant.

The prognostic value for relapse-free survival (RFS) of survival-related DEGs was tested with the Kaplan–Meier Plotter tool, which includes mRNA gene chip data of 7830 breast cancer patients across 55 independent databases [23]. The analyses were performed following the instructions, and the inclusion criteria for patients selection were: ER+ (IHC)/HER2− (array), had at least 10 years follow-up, received adjuvant systemic treatment (the menopausal status was unavailable). The survival-related DEGs were tested both as a whole signature (the mean expression of DEGs was calculated) and as separate genes.

### 2.6. Statistical Analysis

PAM50 subtype scores were compared with the Mann–Whitney test and plotted in GraphPad PRISM 5 (GraphPad Software, Inc., San Diego, CA, USA). Kaplan–Meier curves were fitted and plotted using the “survfit” and “ggsurvplot” function in R, respectively. The cut point was the median of the observed gene expression or signature data. Other statistical analyses were performed using SPSS 23.0 software (IBM, Armonk, NY, USA). All significance tests (where applicable) were two-tailed. Univariate and multivariate survival analyses were performed using the Cox proportional hazards regression model. Statistically significant variables estimated in univariate analyses were included in the multivariate analysis. Spearman correlations were computed between signatures. A *p*-value < 0.05 was considered significant.

## 3. Results

### 3.1. Patient Characteristics and Treatment

A total of 97 premenopausal patients diagnosed with primary HR+/HER2− EBC were included in our cohort. Follow-up in patients who did not develop metastases (M0 group, *n* = 49) was 121–191 months (median: 149 months). DMFS (distant metastasis-free survival) in patients who developed metastases (M1 group, *n* = 48) was between 7 and 184 months (median: 54 months). The most frequent metastasis sites were bone (33/48; 69%), liver (16/48, 33%), lung (9/48, 19%), brain (5/48, 10%), and lymph nodes (5/48, 10%). Clinical patient characteristics (age, tumor size, side, grade, histological type, pT (pathological tumor stage), pN (pathological node status), and treatment strategies) are summarized in Table 1. Patients received treatment according to applicable guidelines: all patients received surgery (lumpectomy or mastectomy) and endocrine therapy (tamoxifen or aromatase inhibitor or sequence of both). In addition, most of the patients received radiotherapy (85.6%) and chemotherapy (72.2%) (the specific regimen varied) (Table 1).

### 3.2. Expression Profiles of Premenopausal HR+/HER2− EBC with Subsequent Metastasis Are Associated with More Aggressive Molecular Subtypes

In order to elucidate mechanisms that contribute to metastatic processes and to identify prognostic metastasis signatures and markers in premenopausal HR+/HER2− EBC, comparative expression analyses were performed between metastatic and non-metastatic tumors.

**Figure 1:**
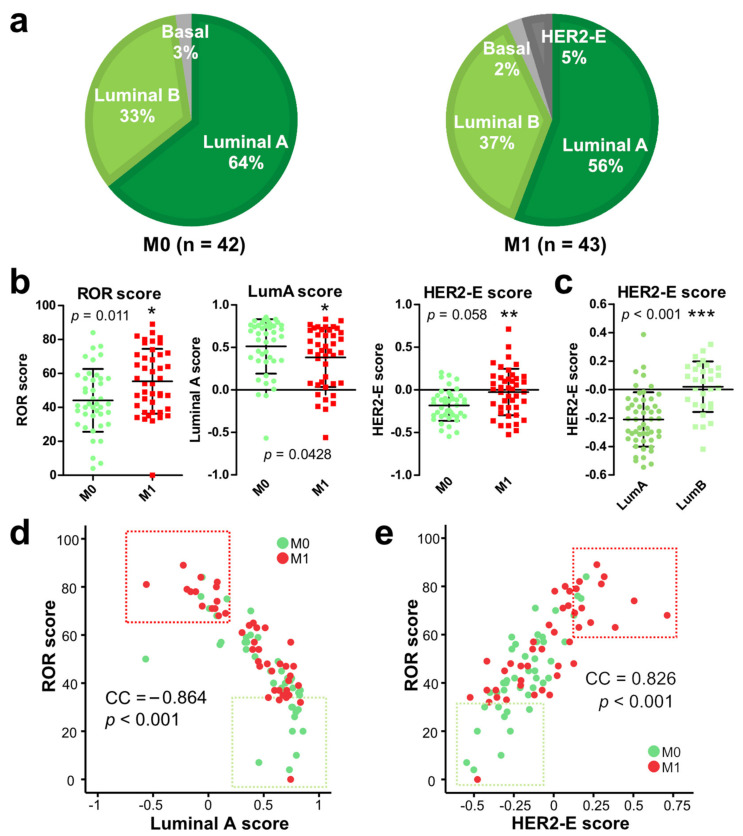
Molecular subtypes in premenopausal breast cancer patients with (M1) and without (M0) subsequent metastasis. (**a**) Proportion of patients in M0 and M1 groups with the indicated molecular subtypes. (**b**) Comparison of Luminal A, HER2-E, and ROR scores between M0 and M1 groups. *, *p* < 0.05, **, *p* < 0.01, ***, *p* < 0.001. (**c**) Comparison of HER2-E score between Luminal A (LumA) and Luminal B (LumB) groups. (**d**,**e**) Correlation of ROR score and Luminal A (**d**) or HER2-E (**e**) score. CC, Spearman correlation coefficient. *p* values are indicated.

An initial molecular subtype analysis (PAM50) showed that 95.3% (81/85) of the tumors displayed luminal status. Compared to the M0 group, the M1 group had a lower proportion of luminal A subtypes (M0, 64%; M1, 56%) and a higher proportion of Luminal B subtypes (M0, 33%; M1, 37%) (Figure 1a). However, the differences were not statistically significant.

As explained above, PAM50 subtyping actually provides a score for each molecular subtype (luminal A, luminal B, HER2-E, basal). These data enabled an analysis of whether subtype scores (not just calls) were quantitatively associated with metastatic onset. Indeed, in our premenopausal collective, ROR scores were significantly higher in M1 patients (*p* = 0.01), which was consistent with the established ROR score application in post-menopausal patients. Luminal A scores were lower (*p* = 0.04) and HER2-E scores higher in M1 patients (*p* = 0.006) (Figure 1b). Moreover, HER2-E scores were also significantly higher in luminal B than luminal A specimens (*p* < 0.001) (Figure 1c). Of note, ROR scores were negatively correlated with luminal A scores (correlation coefficient (CC): −0.864; *p* < 0.001) (Figure 1d) and positively correlated with HER2-E score (CC: 0.826; *p* < 0.001) (Figure 1e). This suggests that the expression profiles in tumors that form metastasis were associated with more aggressive molecular subtypes in premenopausal HR+/HER2− EBC.

### 3.3. Increased ROR Scores and MTORC1 Signaling as Well as Reduced Claudin-Low, Mammary Stemness and PGR Signatures Are Associated with Premenopausal EBC with Subsequent Metastasis

**Figure 2:**
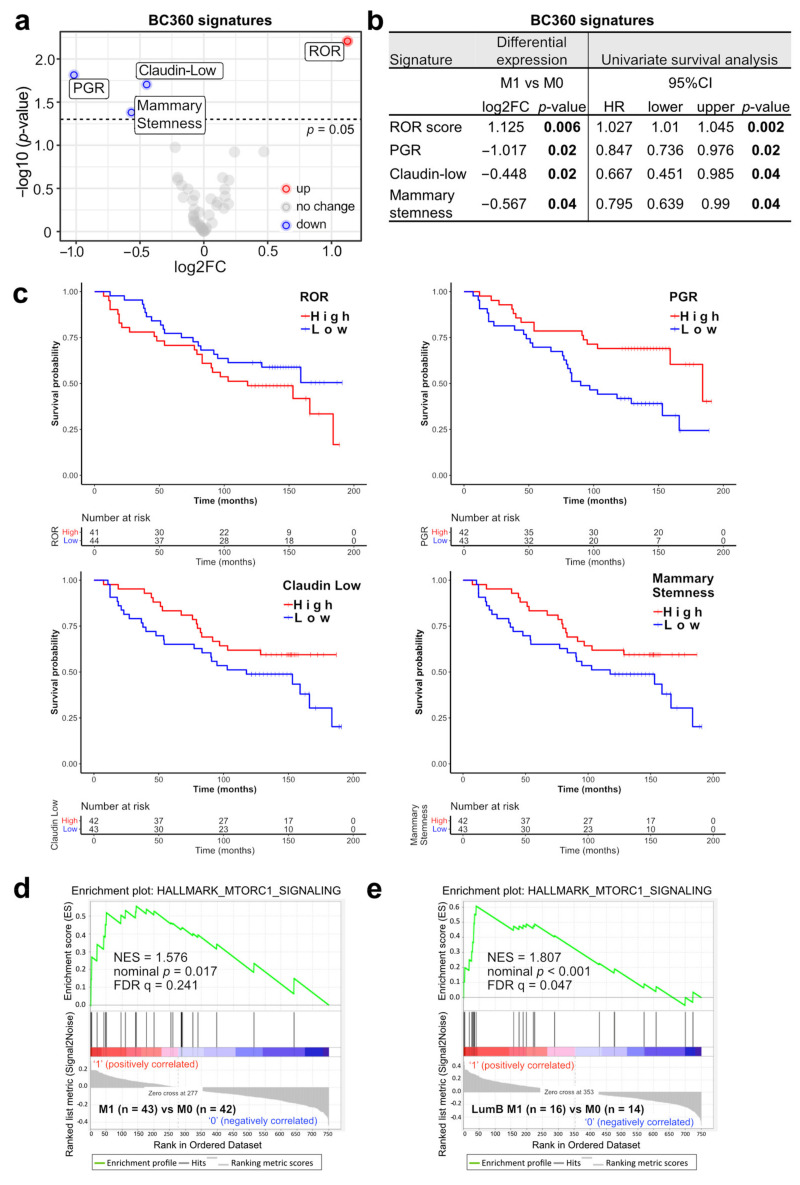
BC360 signatures differentially expressed in M0 vs. M1 are associated with survival. (**a**) Volcano plot of differentially expressed BC360 signatures. Log2 FC, Log2 fold change. Cutoff: *p*-value = 0.05. (**b**) Univariate survival analysis (calculated with Cox regression model) of significant BC360 signatures. HR, hazard ratio. CI, confidence interval. (**c**) Overall survival curves are stratified by the indicated signatures. (**d**,**e**) Gene set enrichment analysis (GSEA) results of M0 vs. M1 ((**d**), all samples) and M0 vs. M1 in Luminal B samples only (**e**). FDR, false discovery rate, NES, normalized enrichment score.

To continue deciphering changes in premenopausal EBC with and without subsequent metastasis, the scores of 46 BC360 signatures were compared between M0 and M1 patients. Four significantly different signatures were noticed: ROR (*p* = 0.006) was up-regulated in M1 patients, while claudin-low (*p* = 0.04), mammary stemness (*p* = 0.02), and PGR (progesterone receptor) (*p* = 0.02) were down-regulated in M1 patients (Figure 2a,b). Expression of the corresponding signature genes, molecular subtypes, and signature scores for each patient are depicted in Figure S2.

Subsequent univariate survival analysis additionally confirmed the importance of these four signatures as they were also associated with patient DMFS. Lower scores of the claudin-low (*p* = 0.04), mammary stemness (*p* = 0.04), PGR (*p* = 0.02) signatures and higher ROR scores (*p* = 0.002) were related to shorter DMFS (Figure 2b,c). The signature associations with survival were consistent with the corresponding up- or down-regulation in the M1 group. Additional subgroup analyses showed that ROR was significantly associated with distant metastases in luminal A patients (LumA M1, *n* = 24 vs. LumA M0, *n* = 27; *p* = 0.02) and in luminal B patients (LumB M1, *n* = 16 vs. LumA M0, *n* = 14; *p* = 0.04) (Figure S1a). Interestingly, lower PGR expression was only significantly associated with distant metastasis in luminal A patients (*p* = 0.002) but not in luminal B patients (*p* = 0.5). This suggests that downmodulation of PGR in luminal A M1 samples accounts for the observed PGR reduction in all M1 samples.

Moreover, GSEA was carried out to test for associations in additional defined datasets correlated with cancer progression and metastasis. The HALLMARK_MTORC1_SIGNALING gene set was enriched in M1 compared with M0 (normalized enrichment score [NES] = 1.576, FDR q = 0.241) (Figure 2d). Subgroup analyses showed that the enrichment of this gene set was even more significant in luminal B M1 patients (NES = 1.807, FDR q = 0.047) (Figure 2e) and not significant in luminal A M1 patients (NES = 0.9409, FDR q = 0.5421) (Figure S1b). This suggests that the observed enhanced MTORC1 signaling was attributed to metastatic luminal B patient samples.

Taken together, we associated increased ROR scores and MTORC1 signaling as well as reduced claudin-low, mammary stemness, and PGR signatures with HR+/HER2− premenopausal EBC with subsequent metastasis.

### 3.4. SCUBE2, LRP2, and IBSP Are Independent Prognostic Markers of Subsequent Metastasis of Premenopausal HR+/HER2− EBC

**Figure 3:**
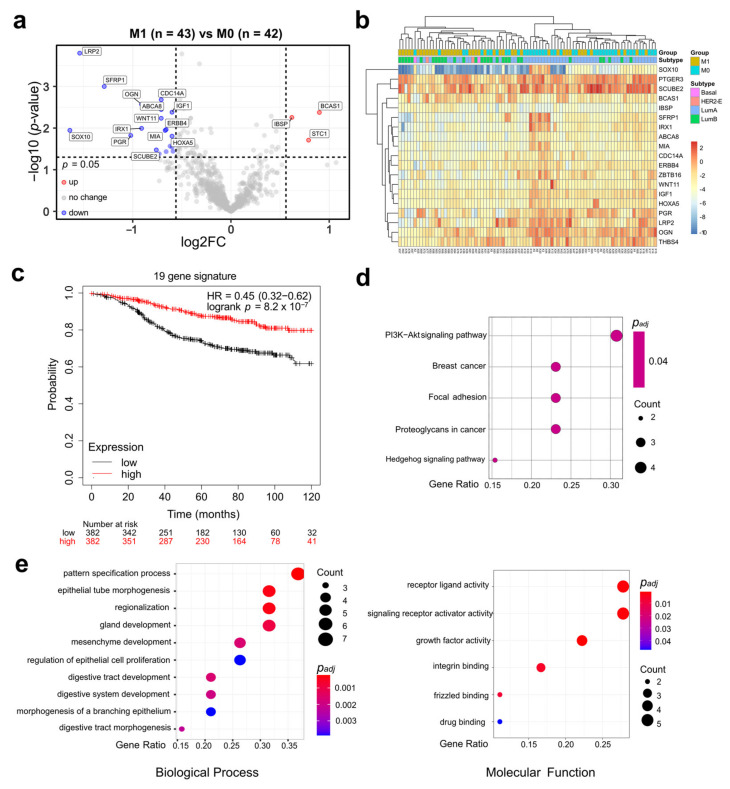
A total of 19 DEGs are differentially expressed in M1 and associated with survival. (**a**) Volcano plot of differentially expressed genes (DEGs). Cutoffs: *p*-value = 0.05, log2 FC = 0.586. Due to limited space THBS4, PTGER3, SFRP4, HSPA2, and ZBTB16 are not labeled. (**b**) Heatmap of DMFS-related 19 DEGs. (**c**) Prognostic value of the 19 DEG signature utilizing various independent datasets available in the Kaplan–Meier Plotter tool. (**d**) Kyoto Encyclopedia of Genes and Genomes (KEGG analysis of DMFS-related 19 DEGs. (**e**) Gene Ontology (GO) analysis of DMFS-related 19 DEGs.

In order to identify a metastasis signature and single prognostic markers for HR+/HER2− premenopausal EBC, comparative analyses were conducted using the expression of all 758 genes. Twenty-two genes passed the cutoff (log2 FC > 0.586 and *p* < 0.05) and were selected for survival analysis. A total of 19 differentially expressed genes (DEGs) were downregulated in M1 samples: *LRP2*, *SFRP1*, *CDC14A*, *OGN*, *ABCA8*, *IGF1*, *WNT11*, *IRX1*, *ERBB4*, *SOX10*, *MIA*, *PGR*, *HOXA5*, *THBS4*, *PTGER3*, *SCUBE2*, *SFRP4*, *HSPA2*, *ZBTB16*. Three DEGs were up-regulated: *BCAS1*, *IBSP*, and *STC1* (Figure 3a). Detailed gene information and fold changes are displayed in Table 2. *MIA*, *PGR*, and *SFRP1* are also part of the ROR signature (Figure S3).

Of the identified 22 DEGs, 19 DEGs (all except *SFRP4* (*p* = 0.07), *HSPA2* (*p* = 0.06) and *STC1* (*p* = 0.05)) were significantly associated with DMFS in univariate survival analyses (Table 2, Figure 3b). The association of each gene with survival was consistent with their corresponding up- or down-regulation in the M1 group. To validate the prognostic role of the 19 DMFS-related DEGs in ER+/HER2− breast cancer patients in independent datasets Kaplan-Meier Plotter analyses for each gene were performed. The importance of single prognostic markers was confirmed for 15 DEGs (Table 3). Moreover, the expression signature of all 19 DEGs was significantly associated with survival (HR = 0.45; log-rank *p* = 8.2 × 10^−7^ (Figure 3c).

Then we investigated the functional roles of the 19 DEGs with GO and KEGG analyses. The 19 DEGs were significantly associated with several breast cancer-relevant pathways, such as PI3K-Akt (phosphatidylinositol 3-kinase/protein kinase B) signaling (Figure 3d), various biological processes and molecular functions, including growth factor activity, Wnt signaling (frizzled binding), and drug binding (Figure 3e). Several previously described functional interactions of the 19 DEGs were found by STRING analysis (Figure S4).

**Figure 4:**
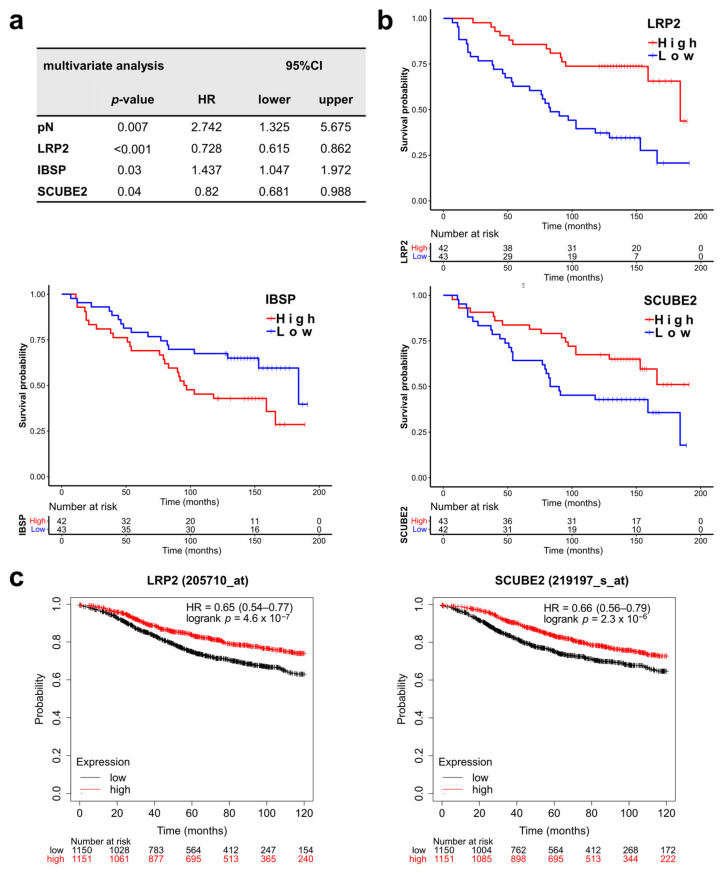
*LRP2*, *SCUBE2*, and *IBSP* are independent prognostic factors. (**a**) Multivariate analysis of differentially expressed BC360 signatures and single genes as well as pN and pT (detailed test results are in Table S2). (**b**) Overall survival curves were stratified by the genes that were significantly associated with survival in the multivariate analysis. (**c**) *LRP2* and *SCUBE2* are prognostic for patients from databases (Kaplan–Meier Plotter). CI, confidence interval. HR, hazard ratio.

Next, we asked which of the significantly associated BC360 signatures and single DEGs represent independent prognostic factors. Therefore, a multivariate survival analysis including pN and pT, four significant signatures (ROR, PGR, claudin-low, mammary stemness), and 22 DEGs were performed. In addition to pN (*p* = 0.007), the single DEGs *LRP2* (*p* < 0.001), *IBSP* (*p* = 0.03) and *SCUBE2* (*p* = 0.04) were identified as independent prognostic factors (Table S2, Figure 4a). Lower *LRP2* and *SCUBE2* levels and higher *IBSP* expression were correlated with shorter DMFS (Figure 4b). The prognostic value of *LRP2* (*p* < 0.001) and *SCUBE2* (*p* <0.001) was confirmed by Kaplan–Meier Plotter analysis in 2301 available patients (Table 3, Figure 4c), whereas only a numerical association was observed for *IBSP* (*p* = 0.13) (Table 3).

In summary, we identified 4 prognostic gene signatures and 19 single-gene markers for distant metastasis in HR+/HER2− premenopausal EBCs that might be applicable in future clinical practice after further confirmation in prospective clinical trials.

## 4. Discussion

For patients with HR+/HER2− EBC, tumor size, tumor stage, nodal status, and tumor grade are widely accepted clinical prognostic factors for making therapy decisions [4]. Besides, risk-stratifying tools based on molecular tests are now useful supplements for risk prediction and treatment planning [7,8]. Nevertheless, most molecular research was performed in post-menopausal patients, and specific evidence for premenopausal patients is still scarce. To facilitate the development of personalized diagnosis and treatment in premenopausal patients, our research investigated molecular prognostic factors for premenopausal patients with HR+/HER2− EBC.

Nearly all tumor samples belonged to luminal subtypes with only four exceptions (two basal and two HER2-E). Luminal A subtype had a higher proportion than luminal B in both M1 and M0 patients, yet the proportion was slightly lower in M1 patients. Though there was no significant difference in subtype calls, the gene expression profile of M1 tumor samples had a closer association with HER2-E molecular patterns and remote association with luminal A molecular patterns than that of M0 tumors. This phenomenon supports the hypothesis of the increased aggressiveness of the M1 tumor samples.

Among the 46 tested BC360 signatures, higher ROR (risk of recurrence) scores, lower scores of PGR (especially in luminal A patients), claudin-low, and mammary stemness were associated with distant metastasis and shorter patient DMFS (distant metastasis-free survival). ROR is frequently used in clinical practice to evaluate post-menopausal patients’ risk of distant recurrence after endocrine therapy [24,25]. However, evidence for the prognostic value of ROR in premenopausal patients is inadequate. Our analysis offers exploratory evidence that ROR could also be useful in evaluating the risk of distant metastasis after standard treatment in premenopausal patients. 

The PGR signature consists of a single gene, namely *PGR*, encoding the Progesterone Receptor (PR), which mediates the physiological effects of progesterone. Low PR expression measured by IHC (immunohistochemistry) is an established indicator for poor prognosis [4]; and higher PR expression is an indicator for better survival [26]. In our study, lower PGR scores were associated with reduced survival among M1 patients (especially in luminal A M1 patients). This suggests that PGR could potentially be used to further differentiate luminal A tumors with a higher risk of metastasis.

The claudin-low signature is defined by low expression of cell-cell adhesion genes, high expression of epithelial-mesenchymal transition (EMT) genes, and stem cell-like/less differentiated gene expression patterns [27]. There are different opinions regarding the prognostic value of the claudin-low subtype [27,28]. Our finding suggests that at least in HR+/HER2− premenopausal EBC, the claudin-low subtype is associated with a good prognosis.

The mammary stemness signature is defined by a cluster of EMT and stem-cell-like related genes. Although previous research suggested that breast cancer stem cells are responsible for tumor aggressiveness, metastasis, and relapse [29], in our patient cohort, a reduced mammary stemness signature was associated with distant metastases and shorter survival. 

To expand our analyses, a comprehensive GSEA analysis was conducted and showed that in premenopausal patients, mammalian target of rapamycin complex 1 (mTORC1) signaling is significantly associated with distant metastases after standard endocrine therapy. Further subgrouping attributed this finding primarily to expression changes in luminal B patients. As a biological characteristic, mTORC1 signaling is involved in many biological processes, including cell cycle progression, growth, and metabolism, and is considered a crucial regulator in health, disease, and aging [30]. Rapamycin and rapalogs (rapamycin derivatives) are well-developed inhibitors of mTORC1 signaling and have various clinical applications, including preventing rejections in transplantations and treating cancers [31]. As a rapalog, everolimus is in clinical use to treat HR+/HER2− BC that are resistant to endocrine therapy in pre- and post-menopausal patients [31,32] (p.79). Besides, second-generation mTOR inhibitors, which include mTOR and PI3K dual-specificity inhibitors and selective mTORC1/2 inhibitors, are under multiple clinical trials to resolve endocrine resistance of HR+/HER2− BC [33,34]. Our report suggested that the enriched mTORC1 signaling in early cancer stages could be an indicator for the development of distant metastases after the current treatment routine (especially in luminal B patients) and early application of mTORC1 inhibitors might be a therapeutic option in this group of patients.

In the next step, we identified a metastasis signature of 19 DEGs that were associated with both metastasis and survival. Lower expression of *LRP2*, *SFRP1*, *CDC14A*, *OGN*, *ABCA8*, *IGF1*, *WNT11*, *IRX1*, *ERBB4*, *SOX10*, *MIA*, *PGR*, *HOXA5*, *THBS4*, *PTGER3*, *SCUBE2*, and *ZBTB16* and higher expression of *BCAS1* and *IBSP* were associated with shorter DMFS. Importantly, the prognostic values of the 19 DEG expression signature and of 15 single genes were also found in other independent ER+/HER2− datasets. Functionally, the 19 DEGs participate actively in several pathways, such as PI3K-Akt (phosphatidylinositol 3-kinase protein kinase B) signaling, which is highly related to the prognosis of breast cancer [4].

Subsequent multivariate analysis including pN, pT, the four identified significant BC360 signatures (ROR, PGR, claudin-low, mammary stemness), and 19 DEGs showed that *LRP2*, *IBSP*, and *SCUBE2* were independent prognostic factors for patients’ DMFS even when compared with clinical prognostic factors. After algorithm development and verification in a larger prospective study, the metastasis signature and single prognostic markers might help to predict metastasis for premenopausal patients.

*LRP2* (LDL receptor related protein 2), a member of the LDL receptor family, is a crucial regulator in the sonic hedgehog pathway, which is important in developmental processes [35]. Higher expression of *LRP2* was reported as a favorable prognostic factor in renal cell carcinoma [36]. Information on *LRP2* in breast cancer is very limited, and its prognostic role was not yet investigated. Our result implies that *LRP2* is also a favorable prognostic factor in premenopausal breast cancer.

*IBSP* (integrin-binding sialoprotein) encodes a secreted glycoprotein that was first discovered in mineralized tissues and subsequently found to be aberrantly expressed in various kinds of malignancies [37]. Increased expression of *IBSP* (also known as *BSP* [bone sialoprotein]) was previously reported to be associated with a higher risk of bone metastasis of breast cancer [37,38]. Our finding stressed the importance of *IBSP* as a potential prognostic marker in premenopausal patients. However, *IBSP* was not associated with survival in the analyses of independent datasets. This might be attributed to the fact that no stratification according to menopausal status was possible.

*SCUBE2* (signal peptide, CUB domain, and EGF-like domain-containing 2) encodes a secreted, membrane-associated multidomain protein, which was reported as a breast tumor suppressor [39] and is included in both MammaPrint^®^ and Oncotype DX^®^, which are widely used diagnostic molecular tests to predict the risk of recurrence [40]. Accordingly, *SCUBE2* was a favorable prognostic factor in our analyses.

The limitations of our study should be noted. Due to the relatively low number of patients in each group, statistical power was limited. Therefore, further group stratification, e.g., by intrinsic subtype and treatment strategy, was not possible. In addition, the number of investigated genes of 770 was also limited due to the Nanostring technology. However, the advantages of our study should not be neglected. Nanostring analyses are very robust and do not need pre-amplification, such as other techniques like RNA sequencing, that might influence measured expression levels. In addition, the analysis failure rate, especially from formalin-fixed tissues, is relatively low when using the Nanostring system. Finally, the evidence of the prognostic value of the 19 DEG-signature and 15 single DEGs in a large number of patients (not exclusively premenopausal) from independent datasets proved the credibility and clinical application potential of our findings.

In summary, in premenopausal patients with HR+/HER2− EBC, enhanced mTORC1 signaling was related to distant metastasis, and higher ROR score, lower scores of PGR, claudin-low, and mammary stemness were risk factors for poor outcomes. Besides, the prognostic genes we discovered (especially *LRP2*, *IBSP*, and *SCUBE2*) may be suitable to contribute to the optimization of risk-stratifying tools for premenopausal patients.

## Figures and Tables

**Table 1:** Patient information.

Feature/Treatment	Groups	M0 (*n* = 49)		M1 (*n* = 48)	
Age at diagnosis (years)	Median (range)	47 (29–50)		43 (30–50)	
Tumor size (cm)	Median (range)	1.7 (0.3–8.0)		2.3 (0.2–6.3)	
Side	left	22	52.4%	20	46.5%
	right	20	47.6%	23	53.5%
Grade	1	6	12.2%	1	2.1%
	2	31	63.3%	26	54.2%
	3	12	24.5%	21	43.8%
Histological type	ductal	39	92.9%	41	95.3%
	lobular	3	7.1%	2	4.7%
pT	1	31	63.3%	17	35.4%
	2	13	26.5%	25	52.1%
	3	5	10.2%	6	12.5%
pN	0	32	65.3%	13	27.1%
	1	11	22.4%	19	39.6%
	2	4	8.2%	11	22.9%
	3	2	4.1%	5	10.4%
Surgery	lumpectomy	35	71.4%	31	64.6%
	mastectomy	14	28.6%	17	35.4%
ALND	yes	24	49.0%	43	89.6%
	no	25	51.0%	5	10.4%
Radiotherapy	yes	44	100.0%	39	86.7%
	no	0	0.0%	6	13.3%
Chemotherapy	yes	29	60.4%	41	89.1%
	no	19	39.6%	5	10.9%
Taxane	yes	11	47.8%	23	62.2%
	no	12	52.2%	14	37.8%
Antracycline	yes	22	100.0%	32	86.5%
	no	0	0.0%	5	13.5%
Endocrine therapy	TAM	33	75.0%	31	81.6%
	AI + GnRHa	3	6.8%	2	5.3%
	sequence of both	8	18.2%	5	13.2%

M0: no metastasis, M1: metastasis, pT: pathological tumor stage, pN: pathological node status, ALND: axillary lymph node dissection, TAM: tamoxifen, AI: aromatase inhibitor, GnRHa: gonadotropin-releasing hormone agonist.

**Table 2:** Differentially expressed genes (LIMMA test) and survival associations thereof (univariate analysis; calculated with Cox regression model).

Gene Symbol	Gene Description	M1 vs. M0		Univariate Survival Analysis			
						95% CI	
		Log2 FC	*p*-value	HR	*p*-value	lower	upper
*LRP2*	low-density lipoprotein receptor-related protein 2	−1.54	<0.001	0.693	<0.001	0.583	0.823
*SFRP1*	secreted frizzled-related protein 1	−1.29	0.001	0.749	0.002	0.625	0.899
*CDC14A*	cell division cycle 14A	−0.71	0.002	0.623	0.005	0.448	0.867
*OGN*	osteoglycin	−0.91	0.003	0.757	0.008	0.616	0.93
*ABCA8*	ATP binding cassette subfamily A member 8	−0.71	0.004	0.697	0.005	0.54	0.899
*IGF1*	insulin-like growth factor 1	−0.6	0.004	0.682	0.01	0.503	0.925
*BCAS1*	breast carcinoma amplified sequence 1	0.9	0.004	1.33	0.009	1.074	1.648
*IBSP*	integrin binding sialoprotein	0.62	0.006	1.495	0.006	1.122	1.992
*WNT11*	Wnt family member 11	−0.71	0.006	0.705	0.01	0.537	0.928
*IRX1*	iroquois homeobox 1	−0.91	0.01	0.767	0.01	0.625	0.94
*ERBB4*	erb-b2 receptor tyrosine kinase 4	−0.66	0.01	0.803	0.03	0.661	0.975
*SOX10*	SRY-Box Transcription Factor 10	−1.64	0.01	0.882	0.02	0.793	0.982
*MIA*	melanoma inhibitory activity	−0.67	0.01	0.676	0.01	0.495	0.923
*PGR*	Progesterone Receptor	−1.02	0.02	0.847	0.02	0.736	0.976
*HOXA5*	homeobox A5	−0.6	0.02	0.74	0.04	0.56	0.979
*STC1*	stanniocalcin 1	0.79	0.02	1.187	0.052	0.998	1.412
*THBS4*	thrombospondin 4	−0.62	0.03	0.791	0.048	0.627	0.998
*PTGER3*	prostaglandin E receptor 3	−0.6	0.03	0.778	0.02	0.625	0.967
*SCUBE2*	Signal Peptide, CUB Domain, and EGF Like Domain Containing 2	−0.76	0.03	0.844	0.04	0.716	0.994
*SFRP4*	secreted frizzled-related protein 4	−0.59	0.04	0.82	0.07	0.66	1.019
*HSPA2*	Heat Shock Protein Family A Hsp70 Member 2	−0.66	0.04	0.817	0.06	0.662	1.007
*ZBTB16*	Zinc Finger and BTB Domain Containing 16	−0.73	0.04	0.822	0.04	0.68	0.993

M1: metastasis, M0: no metastasis, CI: confidence interval, log2 FC: log2 fold change.

**Table 3:** Prognostic values of the 19 DEGs tested in ER+/HER2− breast cancer patients that received adjuvant systemic treatment in independent breast cancer datasets (Kaplan–Meier Plotter tool).

Gene Symbol	Affymetrix ID	Patients in Cohorts	HR (95% CI)	*p*-Value ^1^
*LRP2*	205710_at	2301	0.65 (0.54–0.77)	4.6 × 10^−7^
*SFRP1*	202035_s_at	2301	0.67 (0.57–0.8)	5.6 × 10^−^^6^
*CDC14A*	205288_at	2301	0.91 (0.76–1.07)	0.25
*OGN*	218730_s_at	2301	0.69 (0.58–0.81)	1.4 × 10^−^^5^
*ABCA8*	204719_at	2301	0.69 (0.58–0.82)	2 × 10^−^^5^
*IGF1*	209540_at	2301	0.71 (0.6–0.84)	8.3 × 10^−^^5^
*BCAS1*	204378_at	2301	0.99 (0.84–1.17)	0.91
*IBSP*	207370_at	2301	1.14 (0.96–1.35)	0.13
*WNT11*	206737_at	2301	0.83 (0.7–0.98)	0.032
*IRX1*	230472_at	764	0.67 (0.49–0.91)	0.011
*ERBB4*	206794_at	2301	0.79 (0.66–0.93)	0.0054
*SOX10*	209842_at	2301	0.71 (0.6–0.84)	8.6 × 10^−^^5^
*MIA*	206560_s_at	2301	0.68 (0.58–0.81)	1.3 × 10^−^^5^
*PGR*	208305_at	2301	0.71 (0.6–0.84)	6.7 × 10^−^^5^
*HOXA5*	213844_at	2301	0.82 (0.69–0.97)	0.02
*THBS4*	204776_at	2301	0.85 (0.72–1.01)	0.062
*PTGER3*	208169_s_at	2301	0.81 (0.68–0.96)	0.014
*SCUBE2*	219197_s_at	2301	0.66 (0.56–0.79)	2.3 × 10^−^^6^
*ZBTB16*	205883_at	2301	0.68 (0.57–0.8)	6.9 x 10^−^^6^

HR: hazard ratio; CI: confidence interval; all patients were with ER+/HER2− breast cancer and received adjuvant systemic treatment. ^1^ Logrank *p*-values calculated by the Kaplan–Meier function are presented. The follow-up was set as 120 months.

## Data Availability

The data presented in this study are available on request from the corresponding authors. The data are not publicly available due to restrictions of the institutional IRB statement in concordance to European/German legislation on data restriction.

## Supplementary Materials

The following are available online at https://www.mdpi.com/article/10.3390/jpm11090835/s1, Figure S1: differential expression of BC360 signatures in molecular subgroups of breast cancer and GSEA of the Lum A subgroup, Table S1: tumor content in each tumor FFPE sample, Figure S2: differential expression of genes and scores of the four significant BC360 signatures, Table S2: multivariate analysis of pN, pT, 4 BC360 signatures, and 22 DEGs. Figure S3: overlap between the 22 DEGs and genes in the differentially expressed signatures, Figure S4: functional interactions of the 19 DEGs that are survival-related.
