# Supplementary material for: Molecular Prognostic Factors for Distant Metastases in Premenopausal Patients with HR+/HER2− Early Breast Cancer
Source: J Pers Med. 2021 Aug 25;11(9):835. doi: 10.3390/jpm11090835 (PMC8468490; doi:10.3390/jpm11090835)
Supplement: Supplementary file 1 [file jpm-11-00835-s001.zip › jpm-1318270-supplementary/supplemntary files/Supplementary figures _JPM_V2.1 Ni et al.pdf]

Supplementary Figures

Molecular Prognostic Factors for Distant Metastases in Premenopausal Patients with HR+/HER2- Early Breast Cancer

Hua Ni [1,⁋], Jörg Kumbrink [2,⁋], Doris Mayr [2], Alina Seiler [1], Friederike Hagemann [1], Tom Degenhardt [1], Sabine Sagebiel [2], Rachel Würstlein [1], Ronald Kates [3], Nadia Harbeck [1], and Tanja K Eggersmann [1,4*]

[1] Breast Center, Department of Gynecology and Obstetrics and Comprehensive Cancer Center (CCC)-Munich, the University Hospital of Ludwig Maximilian University of Munich (LMU), Munich, Germany
[2] Institute of Pathology, Faculty of Medicine, Ludwig Maximilian University of Munich (LMU), Munich, Germany
[3] REK consulting, Otterfing, Germany
[4] Department of Gynecological Endocrinology and Reproductive Medicine, University Hospital of Schleswig-Holstein, Luebeck, Germany
[⁋] These authors contributed equally to this work.
[*] Correspondence: Tanja.Eggersmann@med.uni-muenchen.de

Figure S1

Figure S2

Figure S3

Figure S4

Figure S1 Ni et al.

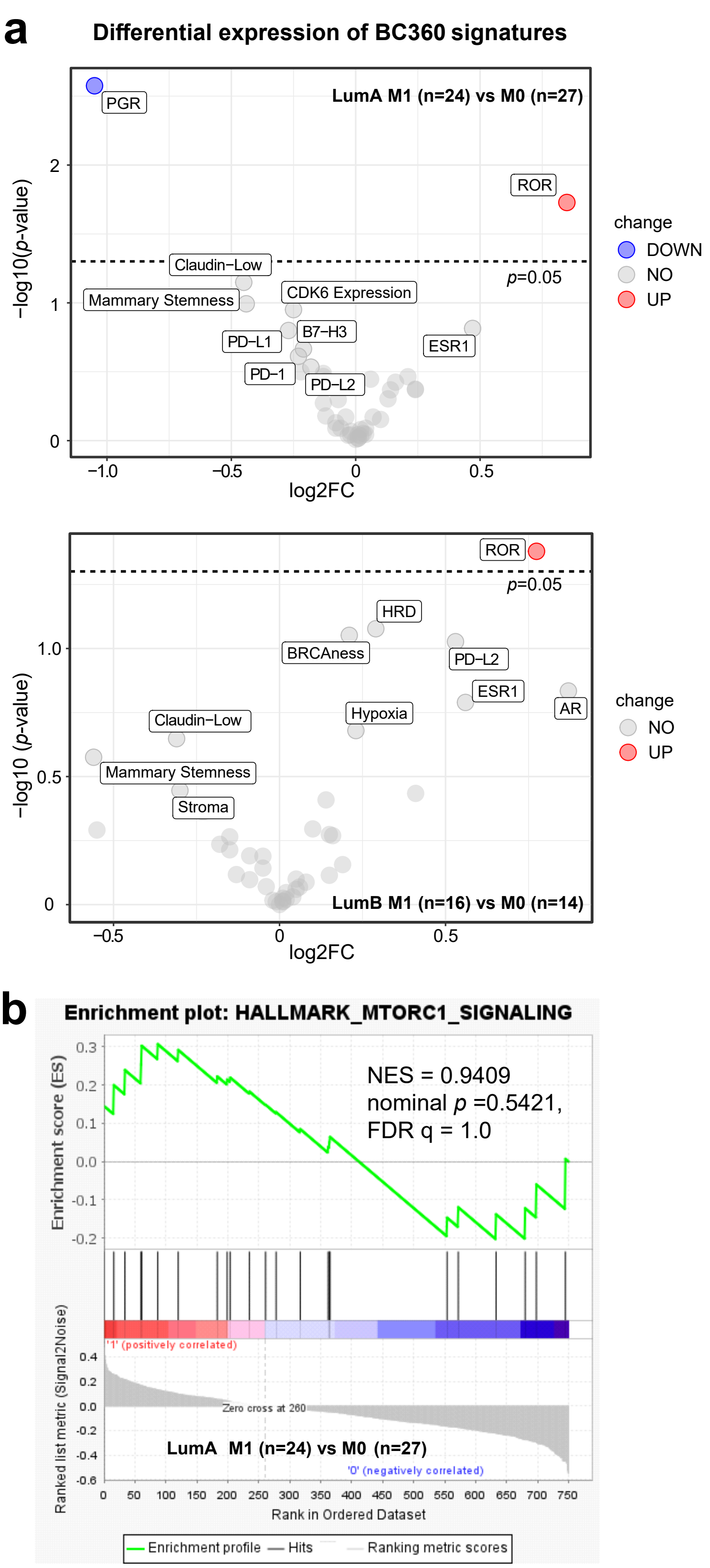

Figure S1. Differential expression of BC360 signatures in molecular subtypes of breast cancer and GSEA of the Lum A subgroup. (a) Volcano plots of differentially expressed BC360 signatures in LumA (M0 vs. M1, upper panel) and LumB (M0 vs. M1, lower panel) tumors. Log_2FC, Log_2 fold change. Cutoffs: *p*-value = 0.05. **(b)** Gene set enrichment analysis (GSEA) results of M0 vs. M1 in Luminal A samples. FDR, false discovery rate, NES, normalized enrichment score.

Figure S2 Ni et al.

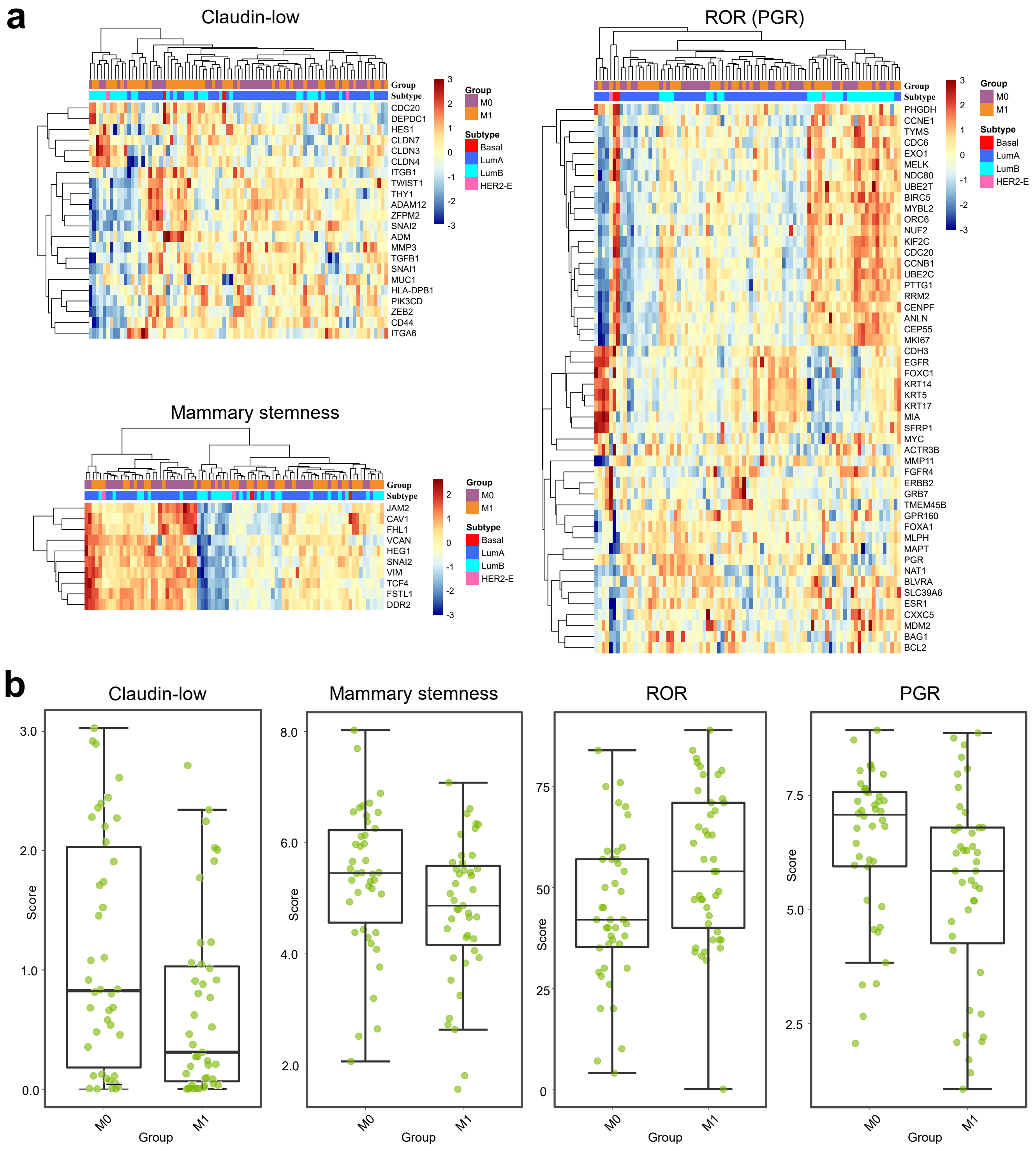

Figure S2. Differential expression of the four significant BC360 signature genes and scores. (a) Heatmaps of the four significantly differentially expressed BC360 signatures (PGR is included in the ROR signature) **(b)** Box plots of the individual scores of relevant BC360 sigantures.

Figure S3 Ni et al.

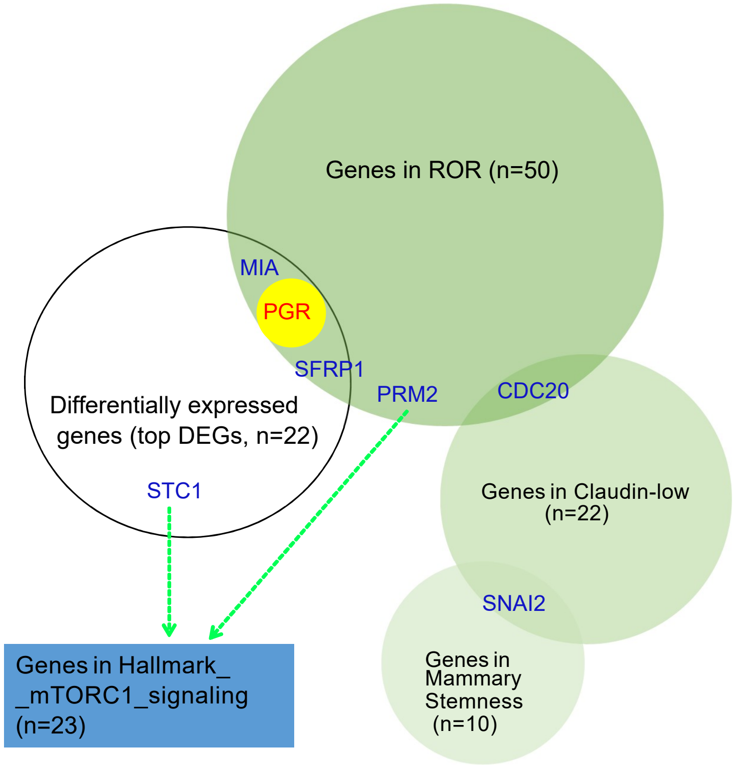

Figure S3. Overlap between the 22 DEGs and genes in the differentially expressed signatures. Red gene names, included in three signatures. Blue gene names, present in two signatures.

Figure S4 Ni et al.

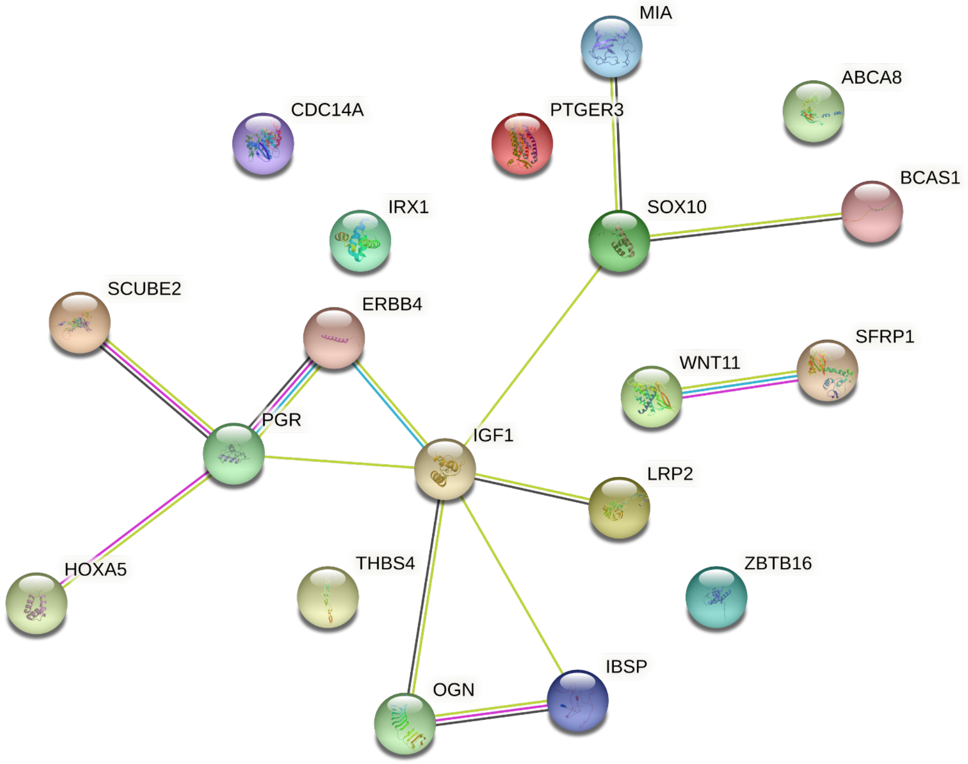

Figure S4. Functional interactions of the 19 DEGs that are survival-related.
