# Supplementary material for: Molecular Prognostic Factors for Distant Metastases in Premenopausal Patients with HR+/HER2− Early Breast Cancer
Source: J Pers Med. 2021 Aug 25;11(9):835. doi: 10.3390/jpm11090835 (PMC8468490; doi:10.3390/jpm11090835)
Supplement: Supplementary file 1 [file jpm-11-00835-s001.zip › jpm-1318270-supplementary/supplemntary files/Table S2 Ni et al.pdf]

Table S2. Multivariate analysis of pN, pT, 4 BC360 signatures and 22 DEGs

			95%CI	
**Factor**	***p*-value**	**HR**	**lower**	**higher**
pN	**0.007**	2.742	1.325	5.675
pT	0.69	-	-	-
PGR	0.20	-	-	-
ROR	0.54	-	-	-
Claudin-low	0.53	-	-	-
Mammary stemness	0.36	-	-	-
LRP2	**<0.001**	0.728	0.615	0.862
SFRP1	0.39	-	-	-
CDC14A	0.17	-	-	-
OGN	0.96	-	-	-
ABCA8	0.93	-	-	-
IGF1	0.58	-	-	-
BCAS1	0.13	-	-	-
IBSP	**0.025**	1.437	1.047	1.972
WNT11	0.21	-	-	-
IRX1	0.57	-	-	-
ERBB4	0.80	-	-	-
SOX10	0.40	-	-	-
MIA	0.14	-	-	-
PGR	0.20	-	-	-
HOXA5	0.80	-	-	-
STC1	0.13	-	-	-
THBS4	0.65	-	-	-
PTGER3	0.24	-	-	-
SCUBE2	**0.037**	0.82	0.681	0.988
SFRP4	0.97	-	-	-
HSPA2	0.62	-	-	-
ZBTB16	0.80	-	-	-

DEG: differentially expressed genes, HR: hazard ratio, CI confidence interval

Forward stepwise regression (likelihood ratio) was used. This method selected the most significant independent prognostic factors to be included into the final equation and calculated the HR of the selected factors.
